# 1-{[(*E*)-(4-{[(2*Z*)-2,3-Di­hydro-1,3-thia­zol-2-yl­idene]sulfamo­yl}phen­yl)iminium­yl]meth­yl}naphthalen-2-olate

**DOI:** 10.1107/S2056989015009640

**Published:** 2015-05-23

**Authors:** Muhammad Shahid, Muhammad Nawaz Tahir, Muhammad Salim, Munawar Ali Munawar, Hazoor Ahmad Shad

**Affiliations:** a Department of Chemistry, University of the Punjab, Lahore, Punjab, Pakistan; b Department of Physics, University of Sargodha, Sargodha, Punjab, Pakistan; c Department of Chemistry, University of Sargodha, Sargodha, Punjab, Pakistan

**Keywords:** crystal structure, zwitterionic compound, sulfa­thia­zole, hydrogen bonding, C—H⋯π inter­actions, π–π inter­actions

## Abstract

In the title zwitterionic compound, C_20_H_15_N_3_O_3_S_2_, the 2-hy­droxy­naphthalene-1-carbaldehyde group *A*, the anilinic unit *B* and the 1,3-thia­zol-2(3*H*)-imine group *C* are each approximately planar with r.m.s. deviation of 0.0721, 0.0412 and 0.0125 Å, respectively. The dihedral angles between *A*/*B*, *A*/*C* and *B*/*C* are 24.70 (10), 79.97 (7) and 83.14 (6)°, respectively. There is an intra­molecular *S*(6) motif involving the imine N—H and the naphtho­late O atom. In the crystal, inversion-related mol­ecules form dimers as a result of N—H⋯N and N—H⋯O hydrogen bonds with *R*_2_^2^(8) and *R*_1_^2^(4) motifs, respectively. Weak π–π inter­actions between the benzene and naphthyl rings of inversion-related mol­ecules have ring centroid–centroid distances of 3.638 (2) and 4.041 (2) Å. A C—H⋯π inter­action occurs between the thia­zol ring and the benzene ring of an adjacent mol­ecule.

## Related literature   

For related structures, see: El-Ghamry *et al.* (2008 ▸); Hebbachi *et al.* (2013 ▸); Zhang (2009 ▸).
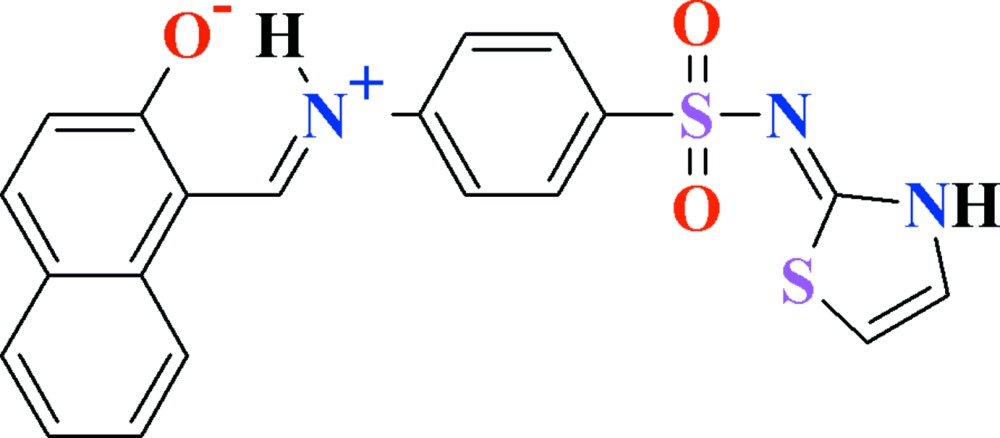


## Experimental   

### Crystal data  

C_20_H_15_N_3_O_3_S_2_
*M* *_r_* = 409.47
Triclinic, 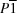

*a* = 9.127 (2) Å
*b* = 10.1417 (12) Å
*c* = 11.355 (3) Å
α = 114.526 (6)°
β = 91.556 (5)°
γ = 102.044 (5)°
*V* = 927.5 (3) Å^3^
*Z* = 2
Mo *K*α radiation
μ = 0.31 mm^−1^
*T* = 296 K
0.32 × 0.26 × 0.18 mm

### Data collection  

Bruker Kappa APEXII CCD diffractometer
Absorption correction: multi-scan (*SADABS*; Bruker, 2005 ▸) *T*_min_ = 0.910, *T*_max_ = 0.948
13121 measured reflections
3554 independent reflections
2086 reflections with *I* > 2σ(*I*)
*R*_int_ = 0.052

### Refinement  

*R*[*F* ^2^ > 2σ(*F* ^2^)] = 0.049
*wR*(*F* ^2^) = 0.127
*S* = 1.01
3554 reflections
253 parameters
H-atom parameters constrained
Δρ_max_ = 0.23 e Å^−3^
Δρ_min_ = −0.25 e Å^−3^

### 

Data collection: *APEX2* (Bruker, 2007 ▸); cell refinement: *SAINT* (Bruker, 2007 ▸); data reduction: *SAINT*; program(s) used to solve structure: *SHELXS97* (Sheldrick, 2008 ▸); program(s) used to refine structure: *SHELXL2014*/7 (Sheldrick, 2015 ▸); molecular graphics: *ORTEP-3 for Windows* (Farrugia, 2012 ▸) and *PLATON* (Spek, 2009 ▸); software used to prepare material for publication: *WinGX* (Farrugia, 2012 ▸) and *PLATON*.

## Supplementary Material

Crystal structure: contains datablock(s) global, I. DOI: 10.1107/S2056989015009640/pk2552sup1.cif

Structure factors: contains datablock(s) I. DOI: 10.1107/S2056989015009640/pk2552Isup2.hkl

Supporting information file. DOI: 10.1107/S2056989015009640/pk2552Isup3.cml

**Fig. 1.**
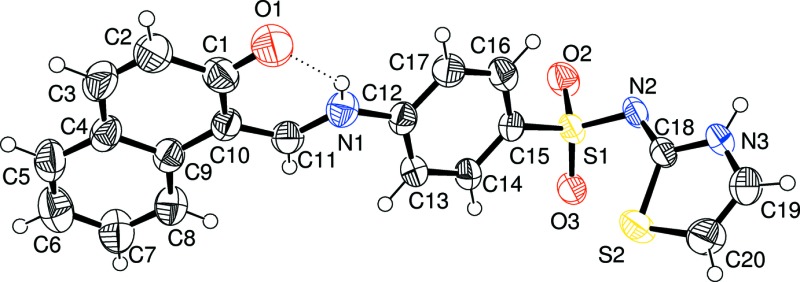
View of the title compound with the atom numbering scheme. Thermal ellipsoids are drawn at the 50% probability level. H-atoms are shown as small circles of arbitrary radius. The dotted lines show intramolecular H-bonding.

. DOI: 10.1107/S2056989015009640/pk2552fig1.tif
View of the title compound with the atom numbering scheme. Thermal ellipsoids are drawn at the 50% probability level. H-atoms are shown as small circles of arbitrary radius. The dotted lines show intra­molecular H-bonding.

**Fig. 2.**
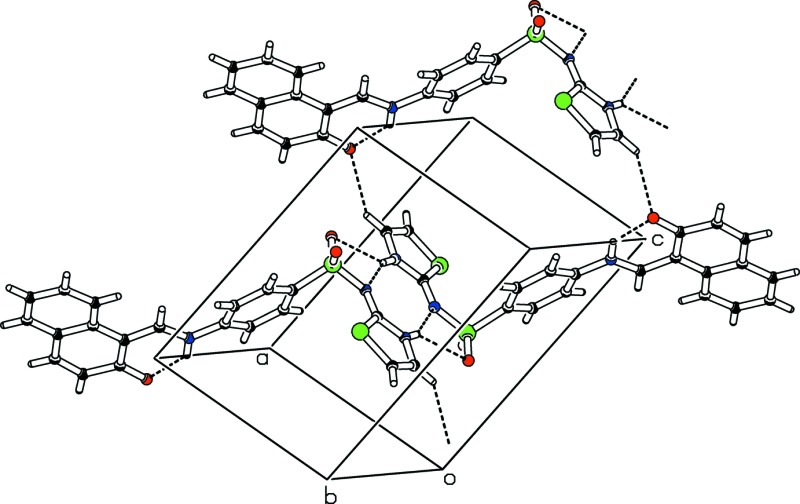
A partial packing plot (PLATON; Spek, 2009), which shows that molecules form dimers and are interlinked forming various ring motifs.

PLATON . DOI: 10.1107/S2056989015009640/pk2552fig2.tif
A partial packing plot (*PLATON*; Spek, 2009), which shows that mol­ecules form dimers and are inter­linked forming various ring motifs.

CCDC reference: 1401829

Additional supporting information:  crystallographic information; 3D view; checkCIF report

## Figures and Tables

**Table 1:** Hydrogen-bond geometry (, ) *Cg*4 is the centroid of the C12C17 ring.

*D*H*A*	*D*H	H*A*	*D* *A*	*D*H*A*
N1H1O1	0.86	1.87	2.550(3)	134
N3H3*A*S1^i^	0.86	2.88	3.729(2)	168
N3H3*A*O2^i^	0.86	2.44	3.131(3)	138
N3H3*A*N2^i^	0.86	2.13	2.943(3)	158
C13H13O2^ii^	0.93	2.60	3.257(4)	128
C19H19O1^iii^	0.93	2.57	3.373(4)	145
C20H20*Cg*4^iv^	0.93	2.99	3.853(4)	156

Symmetry codes: (i) 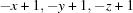; (ii) 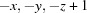; (iii) 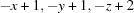; (iv) 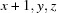.

## supplementary crystallographic information

### S1. Comment

The crystal structures of 1-(4-(diaminomethyleneaminosulfonyl)phenyl iminiomethyl)-2-naphtholate *N*,*N*-dimethylformamide solvate (El-Ghamry, 2008), *N*-(2,3-dihydro-1,3-thiazol-2-ylidene)-4-((2-hydroxybenzylidene)amino) benzenesulfonamide (Zhang, 2009) and 1-(4-((4-((*E*)-(2-hydroxynaphthalen-1-yl) methylideneamino)phenyl)sulfanyl)phenyl)ethanone unknown solvate (Hebbachi, 2013) have been published, and are related to the title compound (I, Fig. 1). (I) was synthesized to study its biological properties and to explore complexation with different metals.

The title compound crystallizes as a zwitterion. In (I), the 2-hydroxynaphthalene-1-carbaldehyde moiety *A* (C1–C11/O1), the anilinic moiety *B* (N1/C12—C17) and the 1,3-thiazol-2(3*H*) -imine group *C* (N2/N3/S1/C18/C19/C20) are planar with r.m.s. deviation of 0.0721, 0.0412 and 0.0125 Å, respectively. The dihedral angles between A/B, A/C and B/C are 24.70 (10)°, 79.97 (7)° and 83.14 (6)°, respectively. The sulfonyl group *D* (S1/O2/O3) is oriented at a dihedral angle of 69.14 (10)° and 55.43 (13)° with *B* and *C*, respectively. There exist intermolecular H-bonding of N—H···O type (Table 1, Fig. 1) forming *S* (6) loop (Bernstein *et al.*, 1995). The molecules are dimerized due to N—H···N type of H-bonding (Table 1, Fig. 2). *R*_1_^2^(4) and *R*_2_^2^(8) rings (Table 1, Fig. 2) (Bernstein *et al.*, 1995) are formed. There exist strong π···π interactions at a distance of 3.638 (2) Å between the centroids of *Cg*2— *Cg*3^i^ and *Cg*3—*Cg*2^i^ [i = - 1 - *x*, - *y*, 2 - *z*], where *Cg*2 and *Cg*3 are the centroids of *E* (C4—C9) and *F* (C1—C4/C9/C10), respectively. Similarly π···π interactions exists between the centeroids of [*Cg*3—*Cg*4^ii^ and *Cg*4—*Cg*3^ii^: ii = - *x*, - *y*, 2 - *z*] at a distance of 4.041 (2) Å. There also exist C—H···π interactions (Table 1). All π···π and C—H···π interactions participate in stabilizing the structure.

### S2. Experimental

Equimolar quantities of 2-hydroxynaphthalene-1-carbaldehyde and 4-amino-*N*-(1,3-thiazol-2-yl)benzenesulfonamide (Sulfathiazole) were refluxed in methanol for 6 h. The solution was kept at room temperature for crystallization which affoarded light orange plates after 72 h.

### S3. Refinement

The H-atoms were positioned geometrically (C–H = 0.93 Å, N—H= 0.86 Å) and refined as riding with *U*_iso_(H) = *xU*_eq_(C, N), where *x* = 1.2 for all H-atoms.

### Crystal data

**Table:**

C_20_H_15_N_3_O_3_S_2_	*Z* = 2
*M**_r_* = 409.47	*F*(000) = 424
Triclinic, *P*1	*D*_x_ = 1.466 Mg m^−^^3^
*a* = 9.127 (2) Å	Mo *K*α radiation, λ = 0.71073 Å
*b* = 10.1417 (12) Å	Cell parameters from 2086 reflections
*c* = 11.355 (3) Å	θ = 2.3–26.0°
α = 114.526 (6)°	µ = 0.31 mm^−^^1^
β = 91.556 (5)°	*T* = 296 K
γ = 102.044 (5)°	Plate, light orange
*V* = 927.5 (3) Å^3^	0.32 × 0.26 × 0.18 mm

### Data collection

**Table:**

Bruker Kappa APEXII CCD diffractometer	3554 independent reflections
Radiation source: fine-focus sealed tube	2086 reflections with *I* > 2σ(*I*)
Graphite monochromator	*R*_int_ = 0.052
Detector resolution: 7.80 pixels mm^-1^	θ_max_ = 26.0°, θ_min_ = 2.3°
ω scans	*h* = −11→11
Absorption correction: multi-scan (*SADABS*; Bruker, 2005)	*k* = −12→10
*T*_min_ = 0.910, *T*_max_ = 0.948	*l* = −13→13
13121 measured reflections	

### Refinement

**Table:**

Refinement on *F*^2^	Primary atom site location: structure-invariant direct methods
Least-squares matrix: full	Secondary atom site location: difference Fourier map
*R*[*F*^2^ > 2σ(*F*^2^)] = 0.049	Hydrogen site location: inferred from neighbouring sites
*wR*(*F*^2^) = 0.127	H-atom parameters constrained
*S* = 1.01	*w* = 1/[σ^2^(*F*_o_^2^) + (0.0596*P*)^2^] where *P* = (*F*_o_^2^ + 2*F*_c_^2^)/3
3554 reflections	(Δ/σ)_max_ < 0.001
253 parameters	Δρ_max_ = 0.23 e Å^−^^3^
0 restraints	Δρ_min_ = −0.24 e Å^−^^3^

### Special details

**Table:**

Geometry. All e.s.d.'s (except the e.s.d. in the dihedral angle between two l.s. planes) are estimated using the full covariance matrix. The cell e.s.d.'s are taken into account individually in the estimation of e.s.d.'s in distances, angles and torsion angles; correlations between e.s.d.'s in cell parameters are only used when they are defined by crystal symmetry. An approximate (isotropic) treatment of cell e.s.d.'s is used for estimating e.s.d.'s involving l.s. planes.
Refinement. Refinement of *F*^2^ against ALL reflections. The weighted *R*-factor *wR* and goodness of fit *S* are based on *F*^2^, conventional *R*-factors *R* are based on *F*, with *F* set to zero for negative *F*^2^. The threshold expression of *F*^2^ > σ(*F*^2^) is used only for calculating *R*-factors(gt) *etc*. and is not relevant to the choice of reflections for refinement. *R*-factors based on *F*^2^ are statistically about twice as large as those based on *F*, and *R*-factors based on ALL data will be even larger.

### Fractional atomic coordinates and isotropic or equivalent isotropic displacement parameters (Å^2^)

**Table:**

	*x*	*y*	*z*	*U*_iso_*/*U*_eq_	
S1	0.26196 (9)	0.20802 (8)	0.46258 (8)	0.0436 (2)	
S2	0.57914 (9)	0.19236 (8)	0.58749 (9)	0.0571 (3)	
O1	−0.0869 (3)	0.3290 (3)	1.1505 (2)	0.0747 (7)	
O2	0.1538 (2)	0.2532 (2)	0.4023 (2)	0.0580 (6)	
O3	0.2907 (2)	0.0638 (2)	0.38835 (19)	0.0498 (5)	
N1	−0.0117 (3)	0.1888 (3)	0.9256 (2)	0.0512 (7)	
H1	0.0035	0.2699	0.9964	0.061*	
N2	0.4105 (3)	0.3410 (2)	0.5032 (2)	0.0449 (6)	
N3	0.6639 (3)	0.4405 (2)	0.5815 (2)	0.0481 (7)	
H3A	0.6652	0.5187	0.5689	0.058*	
C1	−0.1806 (4)	0.2077 (4)	1.1338 (3)	0.0561 (9)	
C2	−0.2866 (5)	0.2126 (4)	1.2278 (4)	0.0728 (11)	
H2	−0.2825	0.3016	1.3007	0.087*	
C3	−0.3895 (4)	0.0895 (4)	1.2094 (4)	0.0691 (10)	
H3	−0.4586	0.0968	1.2692	0.083*	
C4	−0.4008 (4)	−0.0546 (4)	1.1024 (3)	0.0526 (8)	
C5	−0.5088 (4)	−0.1806 (4)	1.0914 (4)	0.0661 (10)	
H5	−0.5771	−0.1709	1.1520	0.079*	
C6	−0.5151 (4)	−0.3180 (5)	0.9924 (4)	0.0789 (11)	
H6	−0.5871	−0.4017	0.9852	0.095*	
C7	−0.4126 (4)	−0.3300 (4)	0.9034 (4)	0.0730 (11)	
H7	−0.4144	−0.4235	0.8372	0.088*	
C8	−0.3080 (4)	−0.2069 (4)	0.9106 (3)	0.0586 (9)	
H8	−0.2418	−0.2186	0.8482	0.070*	
C9	−0.2989 (3)	−0.0644 (3)	1.0099 (3)	0.0471 (8)	
C10	−0.1920 (3)	0.0706 (3)	1.0238 (3)	0.0444 (7)	
C11	−0.1073 (3)	0.0693 (3)	0.9224 (3)	0.0477 (8)	
H11	−0.1189	−0.0206	0.8487	0.057*	
C12	0.0678 (3)	0.1932 (3)	0.8206 (3)	0.0432 (7)	
C13	0.0942 (3)	0.0669 (3)	0.7250 (3)	0.0480 (8)	
H13	0.0687	−0.0233	0.7316	0.058*	
C14	0.1589 (3)	0.0737 (3)	0.6188 (3)	0.0456 (8)	
H14	0.1759	−0.0126	0.5534	0.055*	
C15	0.1990 (3)	0.2078 (3)	0.6081 (3)	0.0402 (7)	
C16	0.1789 (3)	0.3370 (3)	0.7084 (3)	0.0541 (8)	
H16	0.2080	0.4282	0.7039	0.065*	
C17	0.1152 (3)	0.3290 (3)	0.8155 (3)	0.0529 (8)	
H17	0.1042	0.4158	0.8843	0.063*	
C18	0.5396 (3)	0.3322 (3)	0.5515 (3)	0.0396 (7)	
C19	0.7905 (4)	0.4204 (4)	0.6338 (3)	0.0587 (9)	
H19	0.8842	0.4882	0.6581	0.070*	
C20	0.7636 (4)	0.2935 (4)	0.6456 (3)	0.0630 (9)	
H20	0.8354	0.2625	0.6809	0.076*	

### Atomic displacement parameters (Å^2^)

**Table:**

	*U*^11^	*U*^22^	*U*^33^	*U*^12^	*U*^13^	*U*^23^
S1	0.0429 (5)	0.0418 (4)	0.0496 (5)	0.0035 (3)	0.0057 (4)	0.0262 (4)
S2	0.0582 (6)	0.0460 (5)	0.0718 (6)	0.0055 (4)	−0.0039 (4)	0.0340 (4)
O1	0.0905 (19)	0.0571 (14)	0.0641 (16)	0.0136 (14)	0.0200 (13)	0.0156 (12)
O2	0.0507 (14)	0.0628 (13)	0.0693 (15)	0.0066 (11)	−0.0034 (11)	0.0410 (12)
O3	0.0565 (14)	0.0419 (11)	0.0489 (13)	0.0080 (10)	0.0141 (10)	0.0191 (10)
N1	0.0498 (17)	0.0543 (16)	0.0462 (16)	0.0106 (13)	0.0084 (13)	0.0193 (13)
N2	0.0409 (15)	0.0411 (14)	0.0596 (17)	0.0020 (11)	0.0073 (13)	0.0319 (12)
N3	0.0494 (17)	0.0361 (13)	0.0606 (17)	0.0035 (12)	0.0112 (13)	0.0255 (12)
C1	0.068 (2)	0.052 (2)	0.053 (2)	0.0151 (18)	0.0109 (18)	0.0261 (17)
C2	0.100 (3)	0.067 (2)	0.063 (3)	0.033 (2)	0.037 (2)	0.0310 (19)
C3	0.077 (3)	0.090 (3)	0.062 (2)	0.038 (2)	0.035 (2)	0.044 (2)
C4	0.048 (2)	0.078 (2)	0.048 (2)	0.0256 (18)	0.0149 (16)	0.0382 (18)
C5	0.056 (2)	0.093 (3)	0.069 (3)	0.017 (2)	0.0168 (19)	0.054 (2)
C6	0.066 (3)	0.085 (3)	0.087 (3)	−0.003 (2)	0.005 (2)	0.049 (3)
C7	0.077 (3)	0.068 (2)	0.063 (3)	0.004 (2)	0.015 (2)	0.0234 (19)
C8	0.056 (2)	0.065 (2)	0.051 (2)	0.0091 (18)	0.0108 (17)	0.0242 (18)
C9	0.045 (2)	0.062 (2)	0.0433 (19)	0.0161 (16)	0.0071 (15)	0.0295 (16)
C10	0.0411 (19)	0.0555 (19)	0.0433 (19)	0.0162 (15)	0.0104 (15)	0.0254 (15)
C11	0.043 (2)	0.0460 (18)	0.052 (2)	0.0091 (15)	0.0041 (16)	0.0198 (15)
C12	0.0356 (18)	0.0515 (18)	0.0451 (19)	0.0085 (14)	0.0058 (14)	0.0241 (15)
C13	0.046 (2)	0.0468 (18)	0.060 (2)	0.0105 (15)	0.0152 (16)	0.0312 (16)
C14	0.047 (2)	0.0458 (17)	0.049 (2)	0.0104 (14)	0.0159 (15)	0.0252 (15)
C15	0.0320 (17)	0.0415 (16)	0.0461 (18)	0.0048 (13)	0.0063 (13)	0.0198 (14)
C16	0.052 (2)	0.0432 (18)	0.064 (2)	0.0038 (15)	0.0133 (17)	0.0230 (16)
C17	0.054 (2)	0.0410 (17)	0.054 (2)	0.0090 (15)	0.0138 (17)	0.0120 (15)
C18	0.044 (2)	0.0334 (15)	0.0400 (17)	0.0041 (14)	0.0088 (14)	0.0165 (13)
C19	0.042 (2)	0.055 (2)	0.070 (2)	0.0014 (16)	0.0001 (17)	0.0240 (18)
C20	0.048 (2)	0.061 (2)	0.075 (2)	0.0089 (17)	−0.0123 (17)	0.0276 (18)

### Geometric parameters (Å, º)

**Table:**

S1—O2	1.437 (2)	C5—H5	0.9300
S1—O3	1.4373 (19)	C6—C7	1.382 (5)
S1—N2	1.599 (2)	C6—H6	0.9300
S1—C15	1.765 (3)	C7—C8	1.375 (4)
S2—C20	1.726 (3)	C7—H7	0.9300
S2—C18	1.731 (3)	C8—C9	1.400 (4)
O1—C1	1.280 (4)	C8—H8	0.9300
N1—C11	1.323 (3)	C9—C10	1.451 (4)
N1—C12	1.424 (4)	C10—C11	1.401 (4)
N1—H1	0.8600	C11—H11	0.9300
N2—C18	1.322 (3)	C12—C13	1.366 (4)
N3—C18	1.325 (3)	C12—C17	1.381 (4)
N3—C19	1.375 (4)	C13—C14	1.379 (4)
N3—H3A	0.8600	C13—H13	0.9300
C1—C10	1.412 (4)	C14—C15	1.389 (4)
C1—C2	1.453 (4)	C14—H14	0.9300
C2—C3	1.329 (5)	C15—C16	1.386 (4)
C2—H2	0.9300	C16—C17	1.388 (4)
C3—C4	1.442 (4)	C16—H16	0.9300
C3—H3	0.9300	C17—H17	0.9300
C4—C5	1.398 (4)	C19—C20	1.322 (4)
C4—C9	1.408 (4)	C19—H19	0.9300
C5—C6	1.367 (5)	C20—H20	0.9300
			
O2—S1—O3	117.41 (13)	C9—C8—H8	119.3
O2—S1—N2	103.97 (12)	C8—C9—C4	116.5 (3)
O3—S1—N2	112.68 (13)	C8—C9—C10	124.6 (3)
O2—S1—C15	108.18 (13)	C4—C9—C10	118.9 (3)
O3—S1—C15	106.92 (12)	C11—C10—C1	118.7 (3)
N2—S1—C15	107.23 (13)	C11—C10—C9	120.1 (3)
C20—S2—C18	90.63 (15)	C1—C10—C9	121.0 (3)
C11—N1—C12	125.0 (3)	N1—C11—C10	123.7 (3)
C11—N1—H1	117.5	N1—C11—H11	118.1
C12—N1—H1	117.5	C10—C11—H11	118.1
C18—N2—S1	121.34 (19)	C13—C12—C17	120.2 (3)
C18—N3—C19	116.1 (2)	C13—C12—N1	121.6 (3)
C18—N3—H3A	122.0	C17—C12—N1	118.2 (3)
C19—N3—H3A	122.0	C12—C13—C14	119.7 (3)
O1—C1—C10	123.0 (3)	C12—C13—H13	120.2
O1—C1—C2	118.7 (3)	C14—C13—H13	120.2
C10—C1—C2	118.2 (3)	C13—C14—C15	120.9 (3)
C3—C2—C1	119.8 (3)	C13—C14—H14	119.6
C3—C2—H2	120.1	C15—C14—H14	119.6
C1—C2—H2	120.1	C16—C15—C14	119.2 (3)
C2—C3—C4	124.1 (3)	C16—C15—S1	121.3 (2)
C2—C3—H3	118.0	C14—C15—S1	119.4 (2)
C4—C3—H3	118.0	C15—C16—C17	119.4 (3)
C5—C4—C9	121.1 (3)	C15—C16—H16	120.3
C5—C4—C3	121.0 (3)	C17—C16—H16	120.3
C9—C4—C3	117.8 (3)	C12—C17—C16	120.5 (3)
C6—C5—C4	120.7 (3)	C12—C17—H17	119.8
C6—C5—H5	119.6	C16—C17—H17	119.8
C4—C5—H5	119.6	N2—C18—N3	121.3 (3)
C5—C6—C7	118.7 (3)	N2—C18—S2	129.4 (2)
C5—C6—H6	120.6	N3—C18—S2	109.3 (2)
C7—C6—H6	120.6	C20—C19—N3	112.2 (3)
C8—C7—C6	121.4 (3)	C20—C19—H19	123.9
C8—C7—H7	119.3	N3—C19—H19	123.9
C6—C7—H7	119.3	C19—C20—S2	111.8 (3)
C7—C8—C9	121.4 (3)	C19—C20—H20	124.1
C7—C8—H8	119.3	S2—C20—H20	124.1
			
O2—S1—N2—C18	−173.8 (2)	C9—C10—C11—N1	177.4 (3)
O3—S1—N2—C18	−45.6 (3)	C11—N1—C12—C13	−23.2 (4)
C15—S1—N2—C18	71.8 (3)	C11—N1—C12—C17	155.2 (3)
O1—C1—C2—C3	177.0 (3)	C17—C12—C13—C14	−4.5 (5)
C10—C1—C2—C3	0.7 (5)	N1—C12—C13—C14	173.9 (3)
C1—C2—C3—C4	2.6 (6)	C12—C13—C14—C15	0.6 (5)
C2—C3—C4—C5	177.8 (4)	C13—C14—C15—C16	2.6 (4)
C2—C3—C4—C9	−1.8 (5)	C13—C14—C15—S1	−173.1 (2)
C9—C4—C5—C6	2.2 (5)	O2—S1—C15—C16	−60.6 (3)
C3—C4—C5—C6	−177.5 (3)	O3—S1—C15—C16	172.1 (2)
C4—C5—C6—C7	0.0 (6)	N2—S1—C15—C16	51.0 (3)
C5—C6—C7—C8	−1.7 (6)	O2—S1—C15—C14	115.0 (2)
C6—C7—C8—C9	1.2 (6)	O3—S1—C15—C14	−12.4 (3)
C7—C8—C9—C4	0.9 (5)	N2—S1—C15—C14	−133.4 (2)
C7—C8—C9—C10	−179.9 (3)	C14—C15—C16—C17	−1.9 (4)
C5—C4—C9—C8	−2.6 (4)	S1—C15—C16—C17	173.7 (2)
C3—C4—C9—C8	177.1 (3)	C13—C12—C17—C16	5.2 (5)
C5—C4—C9—C10	178.2 (3)	N1—C12—C17—C16	−173.2 (3)
C3—C4—C9—C10	−2.1 (4)	C15—C16—C17—C12	−1.9 (5)
O1—C1—C10—C11	−6.1 (5)	S1—N2—C18—N3	177.9 (2)
C2—C1—C10—C11	170.0 (3)	S1—N2—C18—S2	−2.8 (4)
O1—C1—C10—C9	179.3 (3)	C19—N3—C18—N2	178.6 (3)
C2—C1—C10—C9	−4.6 (5)	C19—N3—C18—S2	−0.9 (3)
C8—C9—C10—C11	11.7 (5)	C20—S2—C18—N2	−178.0 (3)
C4—C9—C10—C11	−169.2 (3)	C20—S2—C18—N3	1.4 (2)
C8—C9—C10—C1	−173.8 (3)	C18—N3—C19—C20	−0.4 (4)
C4—C9—C10—C1	5.3 (4)	N3—C19—C20—S2	1.5 (4)
C12—N1—C11—C10	−175.4 (3)	C18—S2—C20—C19	−1.7 (3)
C1—C10—C11—N1	2.8 (5)		

### Hydrogen-bond geometry (Å, º)

Cg4 is the centroid of the C12—C17 ring.

**Table:**

*D*—H···*A*	*D*—H	H···*A*	*D*···*A*	*D*—H···*A*
N1—H1···O1	0.86	1.87	2.550 (3)	134
N3—H3*A*···S1^i^	0.86	2.88	3.729 (2)	168
N3—H3*A*···O2^i^	0.86	2.44	3.131 (3)	138
N3—H3*A*···N2^i^	0.86	2.13	2.943 (3)	158
C13—H13···O2^ii^	0.93	2.60	3.257 (4)	128
C19—H19···O1^iii^	0.93	2.57	3.373 (4)	145
C20—H20···*Cg*4^iv^	0.93	2.99	3.853 (4)	156

Symmetry codes: (i) −*x*+1, −*y*+1, −*z*+1; (ii) −*x*, −*y*, −*z*+1; (iii) −*x*+1, −*y*+1, −*z*+2; (iv) *x*+1, *y*, *z*.
