# Supplementary figures and images for: 1-{[(E)-(4-{[(2Z)-2,3-Di­hydro-1,3-thia­zol-2-yl­idene]sulfamo­yl}phen­yl)iminium­yl]meth­yl}naphthalen-2-olate
Source: Acta Crystallogr E Crystallogr Commun. 2015 May 23;71(Pt 6):o421–2. doi: 10.1107/S2056989015009640 (PMC4459363; doi:10.1107/S2056989015009640)

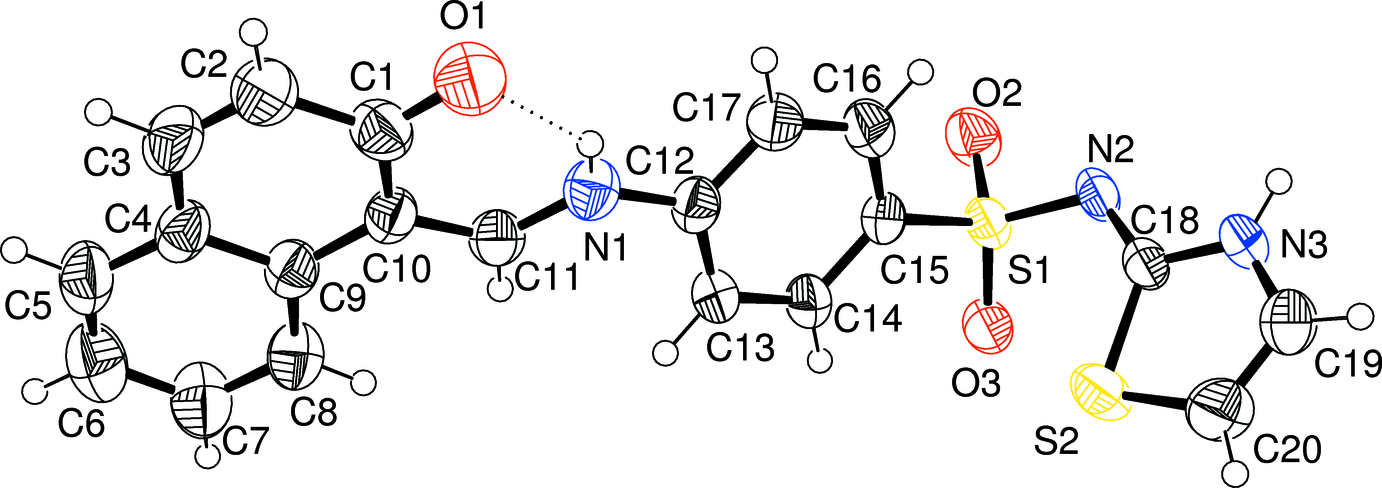

Supplement: Supplementary file 4 [file e-71-0o421-fig1.tif]

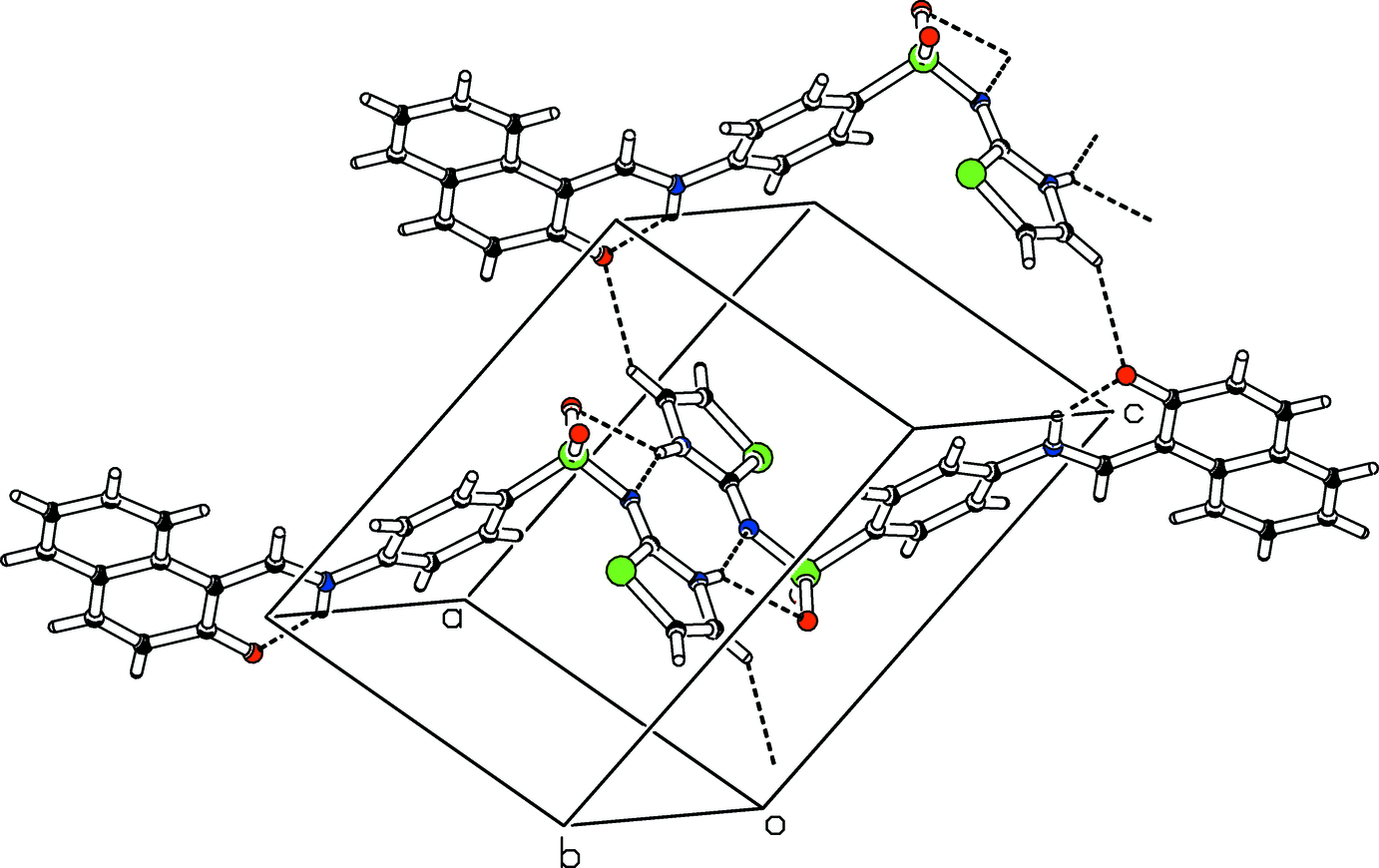

Supplement: Supplementary file 5 [file e-71-0o421-fig2.tif]
